# Optimally Toothed Apertures for Reduced Diffraction

**DOI:** 10.6028/jres.101.072

**Published:** 1996

**Authors:** Eric L. Shirley, R. U. Datla

**Affiliations:** National Institute of Standards and Technology, Gaithersburg, MD 20899-0001

**Keywords:** aperture, computation, diffraction, parallel processing, tooth

## Abstract

We model diffraction errors found when using toothed apertures [L. P. Boivin, Reduction of diffraction errors in radiometry by means of toothed apertures, Appl. Opt. **17**, 3323–3328 (1978)]. Using toothed (cf. circular) apertures minimizes diffraction by inducing destructive interference within the diffracted signal. Since diffraction effects can be quite complicated, their over-all reduction may help limit uncertainties in, say calibrations. Our analysis yields three principles to guide design of nonlimiting (baffle) apertures which minimize diffrac tion. We performed detailed diffraction calculations within scalar (Kirchoff) diffraction theory, using parallel-computing resources at the National Institute of Standards and Technology.

## 1. Introduction

Limiting or nonlimiting apertures (the latter of which are also called baffles) and occulting disks are used in applications ranging from radiometric calibration [1–4] to solar coronagraphy [5]. However, the utility of these devices is hampered by imperfect knowledge of diffraction of radiation at their edges. In radiometry, for instance, diffraction leads to deviation from geometrical optics in the total radiation, from a given source, incident on a detector. Here, we consider the technique of toothing aperture edges to reduce diffraction effects, being motivated by Boivin’s demonstration of the efficacy of this approach [4]. We model Boivin’s experimental results, and we formulate and test three principles to guide design of apertures which lead to minimal diffraction effects. Specifically, we discuss only the effects of teeth on radiation *detected*.

We limit this work to optics having broad-band (e.g., thermal) sources and fully illuminated detectors separated by screens with nonlimiting, (toothed) circular apertures. Diffraction of radiation from broad-band sources is manifested by the total detected flux differing from that predicted by geometrical optics. One desires to know the ratio of actual flux to “geometrical” flux. This ratio is often called “*F*_2_” for optics like those studied here, and “*F*_1_” for certain, analogous optics having limiting apertures [1–4]. As one is concerned with the difference between these ratios and unity, a ratio (*F*_1_ or *F*_2_) may be reexpressed as 1 + ⟨*ε*⟩. This ⟨*ε*⟩ is found by appropriate integration, over wavelength, *λ*, of the relative difference in flux incident for each *λ*, *ε* (*λ*), which could be positive or negative.

In the “*F*_2_” case, Boivin found large effects on ⟨*ε*⟩ from both depth and frequency of teeth, and noted how Huygens’ principle suggested only a tooth’s aspect ratio would affect diffraction. Better analysis of diffraction suggested a minimum necessary tooth depth for a significant reduction in ⟨*ε*⟩. In general, sufficiently deep teeth reduce edge-diffraction effects through path-length-related phase cancellations between diffracted rays created at different points on an aperture perimeter. This destructive phase interference is the key mechanism responsible for Boivin’s observation of a reduction in diffraction effects. However, detailed calculations to model the measured, reduced ⟨*ε*⟩’s were not carried out in Boivin’s presentation.

This paper models the diffraction effects measured by Boivin. The boundary-diffraction-wave formulation [6] helps clarify the effects of depth and frequency of teeth on diffraction. This leads to guiding principles for design of toothed apertures optimal for minimizing diffraction. We test these principles in our own diffraction calculations. Our objectives include understanding the diffraction by toothed apertures, and designing such apertures to control diffraction. This work also demonstrates the feasibility of computing diffraction effects for complex optics, e.g., irregular apertures. We use a parallel-processing implementation of computer programs which model diffraction. Numerical uncertainties in our results are controlled, isolating *numerical* approximations to the scalar Kirchoff theory (which we use) from *physical* approximations of that theory (or, in the case of comparison to measured diffraction effects, experimental errors). In the Kirchoff approach, one evaluates the value of a scalar radiation field behind a screen using the Green’s Function and the value of the undiffracted radiation field *incident* on an aperture.

Below, we first discuss diffraction theory and computational issues pertinent to this work. We next present diffraction calculations modeling Boivin’s ⟨*ε*⟩’s for circular and toothed apertures in 25 optics. Then we identify and apply guiding principles for design of apertures exhibiting minimal diffraction, testing aperture designs within Kirchoff theory by further calculations. We close with some conclusions.

## 2. Diffraction Theory, Computational Issues

Consider (cf. Fig. 1a) an optic consisting of a circular, extended source (radius *ρ*) and fully illuminated, circular detector (radius *r*) placed on opposite sides of a screen with an aperture (nominal radius *R*). Source-screen and screen-detector distances are respectively *a* and *b*. Source, aperture and detector areas lie within parallel planes, and the centers of the areas are colinear, defining an optical axis. *R* is a “nominal” radius only, because we might consider a toothed aperture. The perimeters of toothed apertures are defined as follows. One begins with a circular aperture with radius *R*, and cuts *N* teeth by forming a perimeter consisting of 2*N* straight line segments. At intervals separated by angle *ϕ* = 360°/(2*N*), which is subtended by half of a tooth, the aperture radius alternates between the values, *R* and *R* + *Δ*, where *Δ* is the tooth depth (cf. Fig. 1b).

To evaluate ⟨*ε*⟩, one could perform extensive integration over wavelength and areas of the source, aperture and detector. Use of an “effective-wavelength” [1–3] eliminates wavelength integration, and the boundary-diffraction-wave formulation of Kirchoff theory replaces double integration over aperture area with single integration over its perimeter. Symmetry simplifies integration over either the source or detector. Consider such integration in terms of polar coordinates. For instance, treatment of diffraction by a circular aperture requires no angular integration over the source, and requires angular integration over only half of the detector. Treatment of diffraction by a regularly toothed aperture requires angular integration over only a narrow angular wedge (pie slice) of the source, and integration over only half (or all) of the detector. Integration over the source can be avoided in cases where assuming an axial, point-source is a valid approximation. Then one also needs to integrate flux over only a small part of the detector. We were not able to establish a *predictive* criterion for determining whether this simplification affected results.

Below, we discuss the formula used to describe monochromatic flux incident on the detector, whence a formula for *ε*(*λ*). We also discuss the boundary-diffraction-wave formulation, and the effective-wavelength approximation used in this work, touching finally on considerations regarding the fineness of integration over points on the source, aperture, and detector in the present optics.

We use scalar diffraction theory, so the radiation field, *Ψ*, obeys the Helmholtz equation in free space,

$$[\nabla^{2}+k^{2}]\Psi (r)=0.$$ (1)

Here *k* is the wave number, i.e., 2*π*/*λ*. More specifically, we use Kirchoff theory, which solves for *Ψ* on the detector’s side of the screen using the Green’s Function approach. The Green’s Function is given by

$$G(r,r\prime )=\frac{\exp[ik|r-r\prime |]}{4\pi |r-r\prime |}.$$ (2)

The radiation field at a point ***r***_D_, on the detector (Fig. 1c), is given as

$$\Psi (r_{D})=\int_{\text{aperture}}d^{2}r_{A}\left[\left(\beta +1)G(r_{D},r_{A})\frac{\partial }{\partial n}\Psi_{i}(r_{A})+(\beta -1)\Psi_{i}(r_{A})\frac{\partial }{\partial n}G(r_{D},r_{A}\right)\right].$$ (3)

Here, ***r***_A_ samples all the aperture area. The derivatives are taken normal to the screen plane in the direction towards the detector; *Ψ_i_* is the incident radiation field, not yet specified. The parameter *β* plays the following role: Having *β* = 0 yields the Kirchoff theory; having *β* be +1 or −1 respectively yields extensions of Kirchoff theory which more consistently satisfy *Ψ* = 0 or *∂Ψ*/*∂n* = 0 boundary conditions on the dark side of the screen; cf. Jackson [7] for further discussion of these points.

For a scalar radiation field, intensity is related to a radiation current density,

$$J(r_{D})=\frac{1}{2i}[\Psi^{*}(r_{D})\nabla \Psi (r_{D})-\Psi (r_{D})\nabla \Psi^{*}(r_{D})].$$ (4)

However, for our optics, this is approximately proportional to |*Ψ* (***r***_D_)|^2^. The relative error in the computed intensity, if the latter is estimated using such an approximate value, is 1/*kb*, or typically 10^−7^, because *kb* is typically 10^7^. Therefore, we simply use the square of the radiation field when computing intensity.

If one has an incoherent, extended, broad-band source such as a lamp, detected radiation at each wavelength is often expressed as the sum of fluxes from many monochromatic, mutually incoherent point sources, and we compute our total flux as such a sum. Consider a monochromatic, point source located at point ***r***_S_ on the source. Its radiation field as determined from geometrical optics, which we denote as *Ψ_G_*, is given by the following rule. In the parts of space illuminated by such a source, one has

$${\Psi_{G}(r_{D})|}_{\text{illum}}=\frac{\exp[ik|r_{D}-r_{S}|]}{\left|r_{D}-r_{S}\right|}.$$ (5)

However, in the geometrical shadow of a screen, one has

$${\Psi_{G}(r_{D})|}_{\text{shad}}=0.$$ (6)

In the boundary-diffraction-wave approach, one rewrites the full *Ψ* arising from this point source, including diffraction effects, in this simplified form:

$$\begin{array}{l} \Psi (r_{D})=\Psi_{G}(r_{D})+\\ \int_{\text{aper perim}}dr_{A}\cdot \left[\left(\frac{s\times t}{\mathrm{st}+s\cdot t)}\right)\frac{\exp[ik(s+t)]}{4\pi \mathrm{st}}-\beta \left(\frac{s\times t\prime }{st\prime +s\cdot t\prime }\right)\frac{\exp[ik(s+t\prime )]}{4\pi st\prime }\right]. \end{array}$$ (7)

This is exactly equivalent to the previous formula. Here, ***s*** and ***t*** are respectively vectors from points ***r***_S_ and ***r***_D_ to ***r***_A_; ***t***′ is the vector, to point ***r***_A_, from the point obtained by reflection of ***r***_D_ through the screen plane. These vectors are illustrated in Fig. 1c. For geometries where ***s*** and ***t*** are nearly anti-parallel, the value of *β* is irrelevant. For our optics, this is true; expediency dictates that we set *β* to zero, simplifying diffraction calculations. For later purposes, we make the abbreviation,

$$\Psi (r)=\Psi_{G}(r)+\Psi_{B}(r).$$ (8)

Furthermore, we define *Ψ*_B_(***r***_D_, ***r***_S_; *λ*) as the value of *Ψ*_B_(***r***_D_) arising from a point source at ***r***_S_ emitting radiation at wavelength *λ*; there is an analogous function, *Ψ*_G_(***r***_D_, *λ*).

Next, for a spatially incoherent, monochromatic, extended source, we may write

$$\begin{array}{c} 1+\varepsilon (\lambda )\\ \approx \frac{\int_{\text{source}}d^{2}r_{S}\left[\int_{\text{detector}}d^{2}r_{D}|\Psi_{G}(r_{D},r_{S};\lambda )+\Psi_{B}(r_{D},r_{S};\lambda ){|}^{2}\right]}{\int_{\text{source}}d^{2}r_{S}\left[\int_{\text{dector}}d^{2}r_{D}|\Psi_{G}(r_{D},r_{S};\lambda ){|}^{2}\right]}. \end{array}$$ (9)

The integrand in the numerator may be rewritten as

$$\begin{array}{c} {\left|\Psi_{G}(r_{D},r_{S};\lambda )+\Psi_{B}(r_{D},r_{S};\lambda )\right|}^{2}\\ =|\Psi_{G}(r_{D},r_{S};\lambda ){|}^{2}+{\left|\Psi_{B}(r_{D},r_{S};\lambda )\right|}^{2}\\ +{\Psi^{*}}_{G}(r_{D},r_{S};\lambda )\Psi_{B}(r_{D},r_{S};\lambda )\\ +\Psi_{G}(r_{D},r_{S};\lambda ){\Psi^{*}}_{B}(r_{D},r_{S};\lambda ). \end{array}$$ (10)

Compared to the first (purely geometrical) term, the second term varies roughly as *λ* for optics studied here. For optics in which diffraction of detected radiation implies a substantial angular deflection of that radiation by the aperture, the third (interference) cross-term oscillates rapidly with *λ* and can be largely self-cancelling for broad-band sources. This was found to be the case in the present work.

Such cancellation allows us to use an effective-wavelength approximation, and the particular approximation used here—which differs slightly from earlier effective-wavelength approximations—provides a *λ*-averaged value of *ε*(*λ*), ⟨*ε*⟩, as follows:

$$\langle \varepsilon \rangle =\frac{\int d\lambda \varepsilon (\lambda )S(\lambda )D(\lambda )}{\int d\lambda S(\lambda )D(\lambda )}\approx \varepsilon_{0}(\langle \lambda \rangle ).$$ (11)

For cases when *ε*(*λ*) varies as *λ*, valid in this work, the effective wavelength is

$$\langle \lambda \rangle =\frac{\int d\lambda \lambda S(\lambda )D(\lambda )}{\int d\lambda S(\lambda )D(\lambda )}.$$ (12)

*S*(*λ*) and *D*(*λ*) are the source spectral density and detector responsivity, respectively; *ε*_0_(*λ*) is implied by Eq. (10) after dropping the cross-term. For *ρ* ≪ *a*+*b*, the bracketed integral in the denominator in Eq. (10) depends weakly on ***r***_S_. Ignoring this dependence leads to a relative error of [*ρ*/(*a*+*b*)]^2^, i.e., typically 10^−6^. Therefore, we ignore this dependence. When using Eq. (10) to evaluate ⟨*ε*⟩, or to determine a diffraction pattern for other purposes, one must perform many independent evaluations of *Ψ_B_* (***r***_D_, ***r***_S_; *λ*) and *Ψ*_G_ (***r***_D_, ***r***_D_; *λ*) for different values of ***r***_S_ and ***r***_D_. Use of parallel computing technology can easily accelerate calculations, and we have exploited this fact.

Geometrical and other considerations indicate the required detail in sampling ***r***_S_, ***r***_D_, and ***r***_A_. Ideally, one would conduct only the coarsest sampling necessary. Numerical convergence is improved at the cost of further computation. From calculations which included either coarser or finer samplings than were used to obtain the results presented, we estimate uncertainties, arising from controlled errors in numerical integration, as follows. For each ⟨*ε*⟩, call its expanded uncertainty *δ*⟨*ε*⟩. Then *δ*⟨*ε*⟩/⟨*ε*⟩ is around 0.03 for the results in Table 1 (simulation of Boivin’s experimental numbers), and 0.05 for the results in Table 2 (simulation of a novel design of toothed aperture, to be discussed in a later section), except for results for extended sources, where it is 0.10. These estimates do not include biases related to the effective-wavelength approximation.

We sampled ***r***_D_ at 0.01 mm radial and 0.25° angular intervals, respectively. We improved sampling of ***r***_S_ incrementally, stopping when we could use numerical interpolation to estimate diffracted flux for ***r***_S_ everywhere on the source. Consequently, the radial coordinate of ***r***_S_ was sampled at intervals of 0.05 mm or 0.1 mm. We never found substantial dependence of the diffracted flux on the angular coordinate of ***r***_S_. Symmetry was extensively exploited to accelerate calculations.

Two factors assisted convergence of results with respect to detail of integration. First, the ⟨*ε*⟩’s were sums of strictly positive numbers. Second, distances between various optical components were large compared to components’ dimensions, which were transverse to the optical axes. So phases of emitted or diffracted radiation exhibited only gradual spatial variations over the aperture and detector areas. This facilitated sampling ***r***_A_ at intervals up to 200 times *λ*. Integration about the aperture perimeter in Eq. (7) was usually assisted by two-, four-, or six-point Gaussian quadrature over each line- segment sample of the perimeter. Use of progressively high-ordered Gaussian quadrature permitted diminishing returns of acceleration of the integration.

## 3. Modeling Measured Diffraction Effects

Data modeled were obtained by Boivin [4] using a 3000 K, 1 mm diameter, tungsten-radiation source and RCA 6217 photomultiplier as the detector.^1^ The effective detector area was controlled by 2.5 mm or 3.2 mm diameter proximity apertures. Boivin presented results for at least 27 optics involving one or more intervening apertures. The ⟨*λ*⟩ for this source-detector combination was 0.58 μm. When modeling the data, we shall also analyze the effective-wavelength approximation. Besides that approximation, deviation of our results from experiment can be attributed to Kirchoff theory and/or nonideal experimental circumstances. Experimental difficulties could include misalignments of optical components, even by 0.1 mm (based on our results), or could involve irregularities in aperture perimeters, an effect stressed in Ref. [4].

**Fig. 2:**
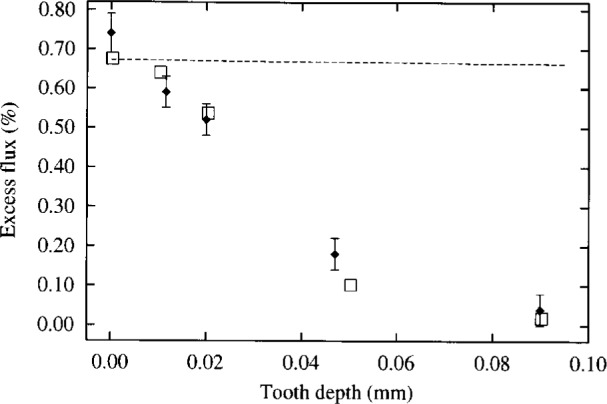
The values of ⟨*ε*⟩ for 7 mm diameter apertures with indicated tooth depth, cf. Configs. 1 to 5 in Table 1. Theoretical points are plotted with squares, experimental points are shown as lozenges. The dashed line shows the theoretical behavior of ⟨*ε*⟩ in the limit of *Δ* approaching zero.

**Fig. 3:**
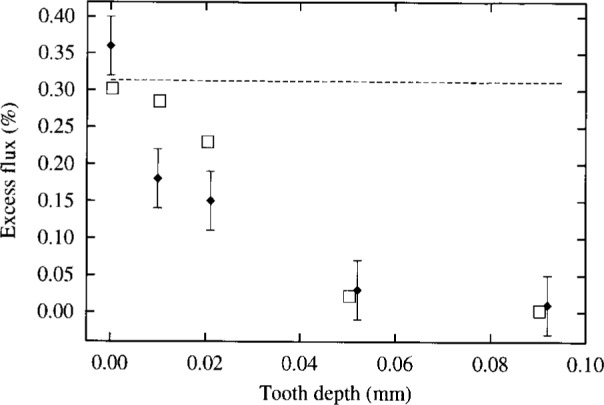
The values of ⟨*ε*⟩ for 15 mm diameter apertures with indicated tooth depth, cf. Configs. 6 to 10 in Table 1. Theoretical points are shown as squares, and experimental points are shown as lozenges. The dashed line shows the theoretical behavior of ⟨*ε*⟩ in the limit of *Δ* approaching zero.

**Fig. 4:**
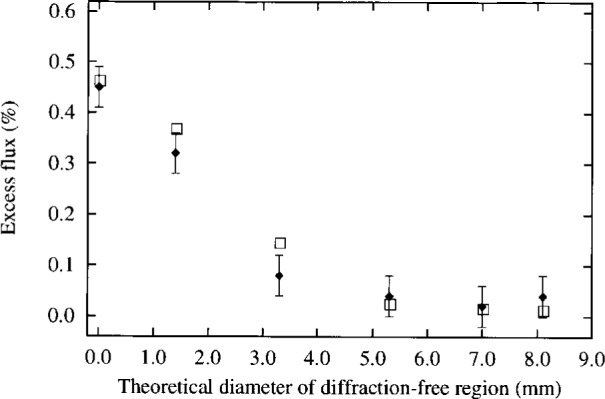
The values of ⟨*ε*⟩ for 7 mm diameter apertures with various numbers of teeth, versus the diameter of a “diffraction-free region,” suggested by a geometrical model in Ref. [4]; cf. Configs. 11 to 16 in Table 1. Theoretical points are shown as squares, and experimental points are shown as lozenges.

**Fig. 5:**
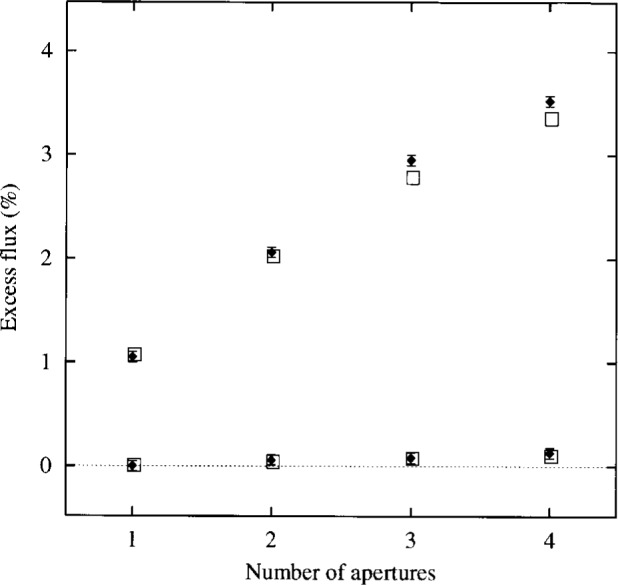
The values of ⟨*ε*⟩ for combinations of apertures from Configs. 17 to 20 measured in Ref. [4], and for combinations of apertures from Configs. 21 to 24, also measured in Ref. [4]. The Configs. are described further in Table 1. Theoretical points are shown as squares, and experimental points are shown as lozenges.

**Fig. 6:**
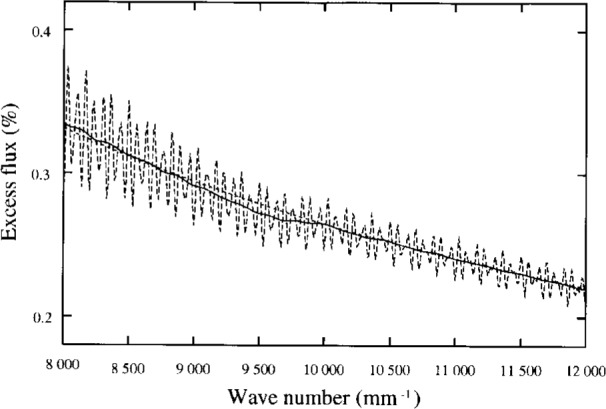
For Config. 8. (cf. Table 1), *ε*_0_(*λ*) (solid line) and *ε*(*λ*) (oscillatory, dashed line), as well as the function 2650/(*k*/mm), shown as a smooth, dashed line, for a range of wave numbers, assuming an axial point source. The apparent irregularities in the theoretical results are genuine.

We computed theoretical ⟨*ε*⟩’s for 25 of Boivin’s optics. Results for combinations of apertures were obtained by adding ⟨*ε*⟩’s computed for the individual apertures separately. (Effects of multiple diffraction by several apertures were assumed to be negligible.) Present results and results by Boivin are tabulated in Table 1. Also shown are parameters specifying optics. The ⟨*ε*⟩’s are given as percentages. We also give the experimental uncertainties in ⟨*ε*⟩, which are indicated in Ref. [4]. Config. 25 in Table 1 involved a 7 mm × 7 mm, square aperture. ***Δ***’s reported in Ref. [4] were up to 0.003 mm different from those used here. This is a negligible effect. In Figs. 2 to 5, we plot results for Config. 1 to Config. 24 in the format of Figs. 3, 6, and 8 of Ref. [4]. In Figs. 2 and 3, we indicate traditional theoretical values of ⟨*ε*⟩ for a round aperture [1–4],

$${\langle \varepsilon \rangle |}_{\text{trad}}\approx \frac{\langle \lambda \rangle b}{\pi^{2}Rr},$$ (13)

as well as extrapolated values valid (within Kirchoff theory) for small ***Δ***,

$${\langle \varepsilon \rangle \approx \langle \varepsilon \rangle |}_{\text{trad}}\left[1-\frac{\Delta }{2R}+O(\Delta^{2})\right].$$ (14)

This latter formula follows from the observation that first-order effects of ***Δ***, those because of a change in average aperture radius, are equivalent to a similar change in the radius of a circular aperture.

## 4. Discussion

We now present results assessing the effective-wavelength approximation, motivate several guiding principles for the design of minimally diffracting apertures, and apply these principles in (simulations of) several “*F*_2_ case” optics.

### 4.1 Effective-Wavelength Approximation

**Fig. 7:**
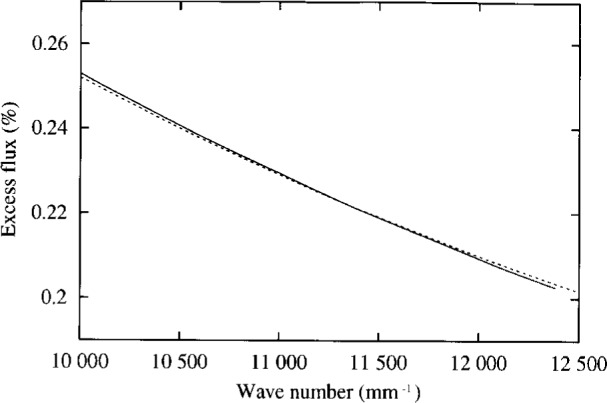
For Config. 8 (cf. Table 1), *ε*_0_(*λ*) (solid line), as well as the function, 2520/(*k*/mm), shown as a dashed line, for a range of wave numbers, assuming an extended source with a 1 mm diameter.

The present effective-wavelength approximation assumes that *ε*_0_(*λ*) is a local average of *ε*(*λ*) with respect to *λ*, and that this local average varies as the first power of *λ*. We have computed *ε*_0_(*λ*) and *ε*(*λ*) for a range of *λ* for Config. 8 in Table 1, but with an axial point source, and we have computed *ε*_0_(*λ*) for a range of *λ* for the same configuration, for a 1 mm diameter, incoherent source. These results are presented in Figs. 6 and 7. Evidently, the effective-wavelength approximation affects results by a small amount of ⟨*ε*⟩ for a point source, and to a lesser degree for an extended source. Selection of Config. 8 for the analysis was spurred by the large discrepancy between its theoretical and experimental ⟨*ε*⟩’s. (Based on these tests of the effective-wavelength approximation, we conclude that such a discrepancy is not attributable to the effective-wavelength approximation.)

### 4.2. Principles for Minimizing Diffraction

Consider an *F*_2_ optic with a circular, toothed aperture, where diffracted radiating reaching the detector would have experienced only a small angular deflection at the aperture perimeter, yet a sufficiently large deflection to permit the present effective-wavelength approximation. Assume that Kirchoff theory is a valid description of diffraction in one’s optic. In calculating *Ψ*_B_(***r***_D_), the vector defining an aperture-perimeter segment, d***r***_A_, has azimuthal and a radial components (cf. Fig. 1b):

$$dr_{A}=\alpha_{1}\hat{u}+\alpha_{2}\hat{\theta }.$$ (15)

(We use cylindrical, polar coordinates, *u*, *θ*, and *z*, and still consider *planar* screens with apertures. Use of *u* for radial coordinate prevents confusing it with source radius, *ρ*.)

When the aperture radius varies azimuthally—e.g., there are teeth—there will be path-length-induced phase differences between diffracted rays, so their contributions to *Ψ*_B_(***r***_D_) by the azimuthal components of the d***r***_A_’s will have large cancellations. Also, these contributions will converge to some limit, if the depth of teeth is fixed, but the frequency of teeth is increased.

Regarding analogous contributions to *Ψ*_B_(***r***_D_) by the ***û***-components of the d***r***_A_’s, there should also be cancellations because of similar effects of phase. Also, cancellations arise from the alternating, inward/outward direction of d***r***_A_. Concomitantly, contributions to *Ψ*_B_(***r***_D_) from the ***û***-components of the d***r***_A_’s will typically exhibit high azimuthal variations, and regularity of *Ψ*_B_ requires contributions with high azimuthal variations to vary as high powers of *u* when *u* is small. Suppose there is a very high frequency of teeth on one*’*s aperture. Then, in the case of a sufficiently remote and/or small source close to the optical axis, a small detector which is also close to the optical axis will receive minimal flux arising from the ***û***-components of the d***r***_A_’s. We confirmed this effect numerically in our calculations.

With the above considerations, two principles of designing baffle apertures that minimize diffraction are as follows:

1. Having deep teeth should help reduce diffracted flux in the central region, because of path-length-induced phase-cancellation effects.
2. Having a high frequency of teeth prevents contributions to the diffracted flux because of radial components of aperture-perimeter segments. This permits one to consider diffracted radiation as the coherent superposition of diffracted radiation from several hypothetical, concentric, circular apertures with different radii. In such a picture, each radius is weighted according to the fraction of the actual aperture perimeter which is at such a radius (distance from the optical axis).

Both of these principles are consistent with the results in Table 1.

For high frequencies of teeth, the diffracted radiation field in the central region is related to a Fourier transform of the radial distribution of the aperture perimeter. Based on Parseval’s theorem, relating the integral of the square of a function to the integral of the square of its Fourier transform, one anticipates that diffraction effects would be smaller for more deeply cut teeth. The diffracted flux is related to the integral, over radius, of the square of the fraction of the aperture perimeter at each value of radius. However, the integral over radius of the first power of that fraction must be one.

One might wish to constrain tooth depth. Very deep teeth may be difficult to realize and, obviously, undermine the functioning of a baffle. Given such a constraint, we propose a third principle for design of minimally diffracting apertures:

(3) Whereas diffracted rays from any single tooth might not be sufficiently weak, diffracted flux on a small, central detector may also be reduced by radially displacing half of the teeth by the distance *πab*/[⟨*k*⟩*R*(*a*+*b*)], ⟨*k*⟩ being 2*π*/⟨*λ*⟩. For diffracted rays reaching the detector, rays originating from one tooth will be similar to, except differing by a 180° phase-shift from, rays originating from an adjacent tooth (cf. Fig. 1d).

The 180° phase-shift depends on the wavelength and above, designed path-length difference. So this third principle works best when one’s source and detector are smaller than an aperture, and the effective bandwidth of radiation transferred for the source-detector combination is small compared to the radiation’s effective central frequency. The 180° phase-shift being exploited would vary as the wave number of radiation from a broad-band source, somewhat degrading the level of destructive interference. The wavelength range having substantial interference would presumably encompass many oscillations with respect to *λ* of the relative signs o the geometrical and boundary-diffraction waves.

To illustrate these three principles further, we consider a set of optics like the others used in this work. We use *a* = *b* = 50 mm, *r* = 1.25 mm, *R* = 7.5 mm, and either an extended source with *ρ* = 0.5 mm or an axial, point source. We vary the number of teeth, *N*, from 120 to 240, 480 and 960, and use several effective wavelengths. Without application of the third principle, we set ***Δ*** = 0.2 mm (cf Fig. 1b). Application of the third principle to aperture design is as follows (cf. Fig. 1d). For the case of *N* teeth, divide an aperture into *N*/4 equal angular wedges. Further, divide each wedge equally into 8 smaller wedges. The aperture perimeter contains one straight segment within each of these smallest wedges. At the nine, equally spaced angles, which collectively define both edges of all eight smallest wedges, the aperture radius has the following values: 7.5 mm, 7.704 84 mm, 7.509 67 mm, 7.714 50 mm, 7.519 34 mm, 7.714 50 mm, 7.509 67 mm, 7.704 84 mm, 7.5 mm. All apertures reflecting the third principle are designed for *λ* = 0.58 mm. So we radially displace alternate pairs of teeth. In Table 2, we present results for ⟨*ε*⟩’s with different combinations of *N*, ⟨*λ*⟩, source, and whether or not the third principle is applied. The results in both Tables demonstrate all three principles, and the third principle is robust with respect to small changes in *λ*.

### 4.3 Miscellaneous Issues

One should critically assess Kirchoff theory and the feasibility for manufacturing and using toothed apertures. This is particularly true for the apertures with the third principle applied, whose design relies on very small structure. One should also consider effects of edge roughness in real apertures. Within Kirchoff theory, sufficiently small features should not have large effects. However, since careful radial displacements of teeth by about 10 mm dramatically influence diffraction, similar irregularities in the apertures of Ref. [4] might contribute to the discrepancy between our ⟨*ε*⟩’s and those reported in this work.

## 5. Conclusions

We have studied the approach of toothing apertures to reduce diffraction. We modeled diffraction effects found by Boivin [4] for 25 optics, each involving an extended, incoherent, broad-band source, one or more apertures, and a fully illuminated detector. Modeled diffraction effects agreed reasonably with those measured, and remaining discrepancies could arise from use of the Kirchoff approach or experimental nonidealities (aperture irregularities, in particular). We also discussed three principles which can guide design of apertures that minimize diffracted flux incident on a central detector. Successful application of these principles was demonstrated, not only in modeling diffraction effects measured by Boivin, but also in simulations of specially designed apertures.

We carried out detailed diffraction calculations using the Kirchoff formulation of scalar diffraction theory, with the work being facilitated by the boundary-diffraction-wave technique. We estimated the level of numerical convergence of these calculations. An effective-wavelength approximation was used, and its validity was assessed. Calculations were accelerated by use of parallel processing, and acceleration of calculations should continue to scale well up to networks of large numbers of processors.

Future research considered includes application of toothed apertures in radiometry, the role of aperture irregularities, further assessments of the effective-wave-length approximation, the validity of the Kirchoff model, and extensions of this work to optics having limiting apertures. One might also consider further simplifying physical and mathematical approximations in the diffraction theory used, as these can reduce computational resources needed for various applications [9].

## Figures and Tables

**Fig. 1(a):**
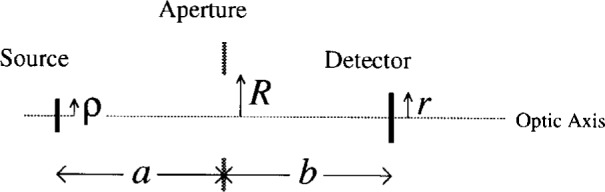
Schematic diagram of *F*_2_ optic: extended source, screen with aperture, and detector; various physical dimensions are indicated.

**Fig. 1(b):**
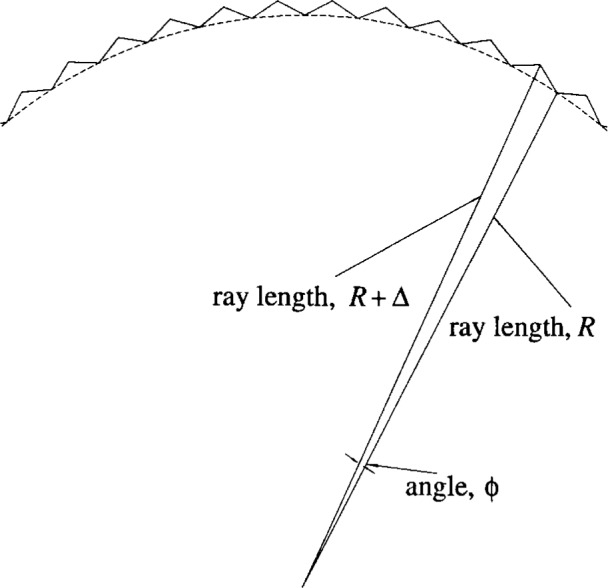
Section of toothed-aperture perimeter.

**Fig. 1(c):**
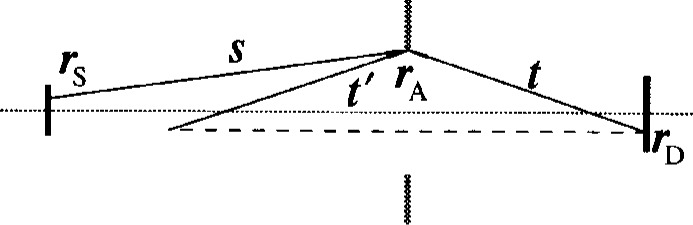
Geometry involved in the boundary-diffraction-wave formulation. Note points on source (***r***_S_), on detector (***r***_D_), on aperture perimeter (***r***_A_), and related vectors, ***s***, ***t***, and ***t***′.

**Fig. 1(d):**
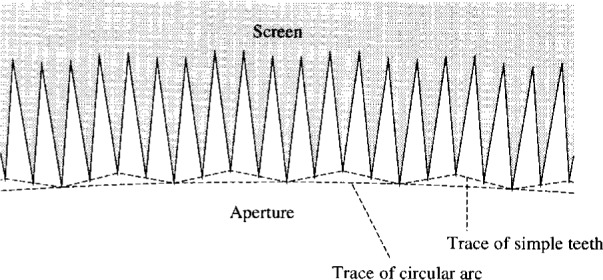
Section of novel, toothed-aperture perimeter.

**Table 1:** Diffraction effects for optical configurations discussed in text

Config.	*a* (cm)	*b* (cm)	*R* (mm)	*Δ* (mm)	*ϕ* (°)	*r* (mm)	⟨*ε*⟩, theory (%)	⟨*ε*⟩, expt. (%)
1	50	50	3.5	smooth		1.25	0.682	0.74(5)
2	50	50	3.5	0.01	3	1.25	0.642	0.59(4)
3	50	50	3.5	0.02	3	1.25	0.541	0.52(4)
4	50	50	3.5	0.05	3	1.25	0.106	0.18(4)
5	50	50	3.5	0.09	3	1.25	0.023	0.04(4)
6	50	50	7.5	smooth		1.25	0.310	0.36(4)
7	50	50	7.5	0.01	3	1.25	0.288	0.18(4)
8	50	50	7.5	0.02	3	1.25	0.234	0.15(4)
9	50	50	7.5	0.05	3	1.25	0.0246	0.03(4)
10	50	50	7.5	0.09	3	1.25	0.0046	0.01(4)
11	85	40	3.5	smooth		1.6	0.464	0.32(4)
12	85	40	3.5	0.1	9	1.6	0.374	0.32(4)
13	85	40	3.5	0.1	5	1.6	0.149	0.08(4)
14	85	40	3.5	0.1	3	1.6	0.026	0.04(4)
15	85	40	3.5	0.1	2	1.6	0.017	0.02(4)
16	85	40	3.5	0.1	1.5	1.6	0.014	0.04(4)
17	50	85	3.5	smooth		1.25	1.10	1.05(5)
18	65	70	3.5	smooth		1.25	0.95	
19	80	55	3.5	smooth		1.25	0.77	
20	95	40	3.5	smooth		1.25	0.57	
17+18			3.5	smooth		1.25	2.05	2.06(5)
17+18+19			3.5	smooth		1.25	2.81	2.95(5)
17+18+19+20			3.5	smooth		1.25	3.38	3.52(5)
21	50	85	3.5	0.1	3	1.25	0.025	0.00(5)
22	65	70	3.5	0.1	3	1.25	0.033	
23	80	55	3.5	0.1	3	1.25	0.035	
24	95	40	3.5	0.1	3	1.25	0.025	
21+22			3.5	0.1	3	1.25	0.058	0.06(5)
21+22+23			3.5	0.1	3	1.25	0.093	0.08(5)
21+22+23+24			3.5	0.1	3	1.25	0.118	0.13(5)
25	50	50	3.5	1.4498	45	1.25	0.13	0.2

**Table 2:** Diffraction for optics discussed in the text.

No. of teeth	Is principle 3 applied?	Source type	*λ* (μm)	⟨*ε*⟩ (%)
120	no	point	0.58	0.000 43
120	yes	point	0.58	0.000 40
240	no	point	0.58	0.000 13
240	yes	point	0.58	0.000 23
480	no	point	0.58	0.000 13
480	yes	point	0.58	0.000 05
960	no	point	0.58	0.000 13
960	yes	point	0.58	0.000 000 43
960	yes	extended	0.71	0.000 009
960	yes	extended	0.58	0.000 008
960	yes	extended	0.49	0.000 017

## Footnotes

1 Certain commercial equipment, instruments, or materials are identified in this paper to foster understanding. Such identification does not imply recommendation or endorsement by the National Institute of Standards and Technology, nor does it imply that the materials or equipment identified are necessarily the best available for the purpose.
